# Supplementary material for: Dietary Intake and the Neighbourhood Environment in the BC Generations Project
Source: Nutrients. 2022 Nov 18;14(22):4882. doi: 10.3390/nu14224882 (PMC9695357; doi:10.3390/nu14224882)
Supplement: Supplementary file 1 [file nutrients-14-04882-s001.zip › nutrients-2027665-supplementary.pdf]

Dietary Intake and the Neighbourhood Environment in the BC Generations Project

Supplementary Material

Supplementary Table S1. Fruit and vegetable consumption by categories of neighbourhood environment

	<5 servings/day, N(%)	≥5 servings/day, N(%)	Mean (SD) servings/day
**Walkability (Can-ALE)**			
Very low	3,018 (41.2)	4,308 (58.8)	5.30 (2.53)
Low	3,690 (41.0)	5,304 (59.0)	5.30 (2.51)
Moderate	2,518 (43.1)	3,319 (56.9)	5.21 (2.68)
High	1,330 (41.9)	1,847 (58.1)	5.22 (2.67)
Very high	1,017 (43.7)	1,310 (56.3)	5.22 (2.96)
**Marginalization (CAN-Marg)**			
Households and dwellings			
Very low	1,593 (41.8)	2,218 (58.2)	5.16 (2.44)
Low	2,479 (41.8)	3,450 (58.2)	5.30 (2.58)
Moderate	2,605 (41.5)	3,678 (58.5)	5.28 (2.51)
High	2,105 (40.6)	3,085 (59.4)	5.28 (2.61)
Very high	2,588 (43.3)	3,385 (56.7)	5.25 (2.80)
Material resources			
Very low	2,196 (42.8)	2,939 (57.2)	5.20 (2.66)
Low	2,893 (43.0)	3,828 (57.0)	5.17 (2.54)
Moderate	2,398 (40.6)	3,514 (59.4)	5.32 (2.61)
High	2,047 (41.2)	2,922 (58.8)	5.32 (2.61)
Very high	1,836 (41.3)	2,613 (58.7)	5.32 (2.59)
Age and labour force			
Very low	3,859 (39.5)	5,909 (60.5)	5.40 (2.63)
Low	3,087 (41.0)	4,445 (59.0)	5.28 (2.54)
Moderate	2,212 (43.5)	2,879 (56.6)	5.19 (2.62)
High	1,554 (46.1)	1,814 (53.9)	5.01 (2.58)
Very high	658 (46.1)	769 (53.9)	5.01 (2.63)
Immigration and visible minority			
Very low	936 (41.5)	1,319 (48.5)	5.27 (2.46)
Low	1,590 (40.4)	2,347 (59.6)	5.35 (2.55)
Moderate	2,391 (39.3)	3,700 (60.8)	5.38 (2.56)
High	3,253 (41.9)	4,505 (58.1)	5.25 (2.59)
Very high	3,200 (44.8)	3,945 (55.2)	5.12 (2.71)
**Material Deprivation**			
Very low	3,389 (38.9)	5,332 (61.1)	5.45 (2.66)
Low	2,746 (41.1)	3,938 (58.9)	5.30 (2.58)
Moderate	2,221 (42.5)	3,005 (57.5)	5.18 (2.50)
High	1,809 (44.6)	2,247 (55.4)	5.11 (2.58)
Very high	1,242 (48.8)	1,303 (51.2)	4.89 (2.59)
**Social Deprivation**			
Very low	2,382 (40.5)	3,499 (59.5)	5.26 (2.48)
Low	2,289 (41.3)	3,257 (58.7)	5.31 (2.55)
Moderate	2,175 (41.8)	3,028 (58.2)	5.28 (2.62)
High	2,297 (42.0)	3,175 (58.0)	5.25 (2.64)
Very high	2,264 (44.1)	2,866 (55.9)	5.19 (2.72)
**Greenness**			
Very low	2,283 (43.8)	2,931 (56.2)	5.18 (2.72)
Low	2,313 (43.5)	3,005 (56.5)	5.26 (2.48)
Moderate	2,732 (44.0)	3,483 (56.0)	5.15 (2.54)
High	2,245 (39.2)	3,479 (60.8)	5.36 (2.58)
Very high	2,212 (38.9)	3,473 (61.1)	5.42 (2.61)
